# Modified Nutrition Risk in Critically ill is an effective nutrition risk screening tool in severely burned patients, compared with Nutrition Risk Screening 2002

**DOI:** 10.3389/fnut.2022.1007885

**Published:** 2022-12-08

**Authors:** Zhenzhu Ma, Yin Zhang, Qin Zhang, Beiwen Wu

**Affiliations:** ^1^Department of Nursing, Ruijin Hospital, Shanghai Jiao Tong University School of Medicine, Shanghai, China; ^2^Department of Burn, Ruijin Hospital, Shanghai Jiao Tong University School of Medicine, Shanghai, China

**Keywords:** severe burns, NRS2002, mNUTRIC, nutrition risk, severity index

## Abstract

**Objective:**

The present study aimed to evaluate the value of Modified Nutrition Risk in Critically ill (mNUTRIC) and Nutrition Risk Screening 2002 (NRS2002) in the prognosis of severely burned patients.

**Methods:**

The retrospective cohort study used medical data of severely burned patients admitted to the burn center of Shanghai Ruijin Hospital between January 2015 and September 2021. Demographics, clinical characteristics, laboratory nutritional indicators, mNUTRIC score and NRS2002 score were collected and analyzed in evaluation the value of two nutrition risk screening tools. Spearman correlation analysis was carried out to show the correlation between variables. The area under receiver operating characteristic (ROC) curve was used to assess the ability of mNUTRIC and NRS2002 to predict mortality. Kaplan–Meier survival curves and log-rank tests were conducted to compare the overall survival (OS). Multivariate Cox proportional hazard regression model was used to identify risk factors for 28-day mortality of severely burned patients.

**Results:**

A total of 429 adult patients with burn area larger than 30% total body surface area (TBSA) were included in this study. Incidence of nutrition risk was detected in 52.21% by mNUTRIC and 20.51% by NRS2002. However, mNUTRIC was superior to NRS2002 in predicting 28-day mortality (area under ROC curve: 0.795 vs. 0.726). Multivariate Cox regression analysis showed that high mNUTRIC [hazard ratio (HR) = 4.265, 95% CI = 1.469–12.380, *P* = 0.008] and TBSA (HR = 1.056, 95% CI = 1.033–1.079, *P* < 0.001) were independent predictors for 28-day mortality. After adjusting for covariates, high NRS2002 was not associated with 28-day mortality (*P* = 0.367).

**Conclusion:**

The present study illustrated the effectiveness of mNUTRIC as nutrition risk screening tool among severely burned patients. Early identification of nutrition risk may help to maximize benefits of nutritional therapy by providing more aggressive nutritional therapy for patients at nutrition risk.

## Introduction

Extensive burns is considered as one of the most serious trauma and can lead to sepsis and metabolic disturbances. A growing body of research has found that these pathophysiological responses in burn patients predispose the body to malnutrition (1, 2). Malnutrition is an independent risk factor for poor prognosis, increased length of stay, mortality, economic cost, and readmission (3–5). However, well-known burn severities are often described as follows: total body surface area (TBSA) and full thickness burn area, inhalation injury. The contribution of nutritional factors to prognosis of burn patients is unclear. It has been proved that nutritional therapy could improve clinical outcomes of critically ill patients (6–8). Because appropriate nutritional therapy plays an increasingly important role in patient care, the Global Leadership Initiative on Malnutrition (GLIM) states that screening critically ill patients for nutrition risk is a critical step in nutritional therapy (9).

Several studies (10–13) applied validated nutrition risk tools and found that patients at nutrition risk are more likely to have adverse outcomes and benefit more from nutritional therapy. Recent guidelines from the Society for Critical Care Medicine (SCCM) and the American Society for Parenteral and Enteral Nutrition (A.S.P.E.N.) recommend the use of the Nutrition Risk Screening 2002 (NRS2002) and Nutrition Risk in Critically ill (NUTRIC) to determine nutrition risk on ICU admission (14). The NUTRIC is a nutrition risk screening tool specially developed for critically ill patients, including age, Acute Physiology and Chronic Health Assessment (APACHE II) score, Sequential Organ Failure Assessment (SOFA) score, number of comorbidities, days from admission to ICU admission and serum interleukin-6 (IL-6) levels (13). The Modified Nutrition Risk in Critically ill (mNUTRIC) simplifies the scoring criteria by removing IL-6, increasing its clinical applicability and the scores for the mNUTRIC vary from 0 to 9. Applying inappropriate screening tools can also result in missed diagnoses or wasted healthcare resources (15). So it is necessary to identify a nutrition risk screening tool that can be adapted to burn patients and determine the prognostic impact of nutrition risk in burn patients.

Although there are many nutrition risk screening tools, to date, there are none developed specifically for burn patients and few have been validated in the population (13, 16, 17). Most of the available nutrition risk screening tools focus on pre-injury dietary and weight loss, and less on the specific metabolic state of burn disease. To date, the prevalence of nutrition risk in burn patients, the comparison of nutrition risk screening tools in burn patients, prognostic impact of nutrition risk have not been extensively studied. The study aims to investigate the prognostic value of nutritional indicators, including mNUTRIC and NRS2002, on the 28-day mortality of severely burned patients.

## Materials and methods

### Study population

A retrospective cohort study design was used to retrospectively collect and analyze the medical records of severely burned patients admitted to the burn center of Shanghai Ruijin Hospital between January 2015 and September 2021. Patients aged older than 18 years, burn area larger than 30% TBSA were enrolled. Patients with incomplete data, readmission for plastic surgery were excluded from this study.

The present study was conducted in compliance with the Declaration of Helsinki and the study protocol was approved by the Research Ethics Committee of Ruijin Hospital, Shanghai Jiao Tong University School of Medicine (Decision No. 2022019). Since this present study was retrospective, patients were not required to provide written informed consent.

### Data collection

Demographic information [age, gender, burn type, body mass index (BMI), and comorbidities], clinical data (TBSA, inhalation injury, APACHE II score, SOFA score, and mortality), and laboratory nutritional parameters [albumin, pre-albumin, neutrophil-to-lymphocyte ratio (NLR), and hemoglobin] were collected from medical records. Outcome measure was defined as 28-day mortality.

### Assessment of nutrition risk

All enrolled patients were screened for nutrition risk by using the mNUTRIC and NRS2002. All nurses received systematic training before they evaluated nutritional status. The nutritional status of the scale entries was entered in Electronic Medical Records (EMR) by the nurses within 24 h of the patient’s emergency admission to the burn center and the scores were calculated by one investigator based on EMR. The high nutrition risk identified by NRS2002 in critically ill patients was defined as NRS2002 score ≥5 (18). Considering different cut-off values of mNUTRIC in previous studies, the Youden’s index was used to determine the cut-off value of mNUTRIC.

### Statistical analysis

Continuous variables are presented as mean ± SD and tested for normal distribution using the Kolmogorov–Smirnov test. Those skewed data were described using the median and inter-quartile range (IQR) and compared using the Mann–Whitney test. Categorical variables were analyzed using Chi-square tests. The Spearman correlation analysis was used to show the correlation between two variables. The receiver operating characteristic (ROC) was used to compare the ability of mNUTRIC and NRS2002 to predict mortality and calculate the best Youden’s index (sensitivity + specificity − 1) to determine the cut-off value. Survival analysis was performed using the Kaplan–Meier method. Multivariate Cox proportional hazard regression model was applied to determine hazard ratios (HRs) for 28-day mortality. Statistical analysis was performed using IBM SPSS 24.0. A *P*-value of <0.05 was considered statistically significant.

### Sample size calculation

The sample size calculation required 80% power at significance level of α = 0.05 to detect a log HRs of logΔ = 1.3863 based on parameters obtained from the first 100 patients in data collection period. The sample size was also adjusted for the *R*^2^ obtained from the regression of all covariates is 0.0094 (19). The required sample size is 404 for a multivariate model. PASS software 15 was used to calculate the sample size.

## Results

### Characteristics of the study population

**FIGURE 1:**
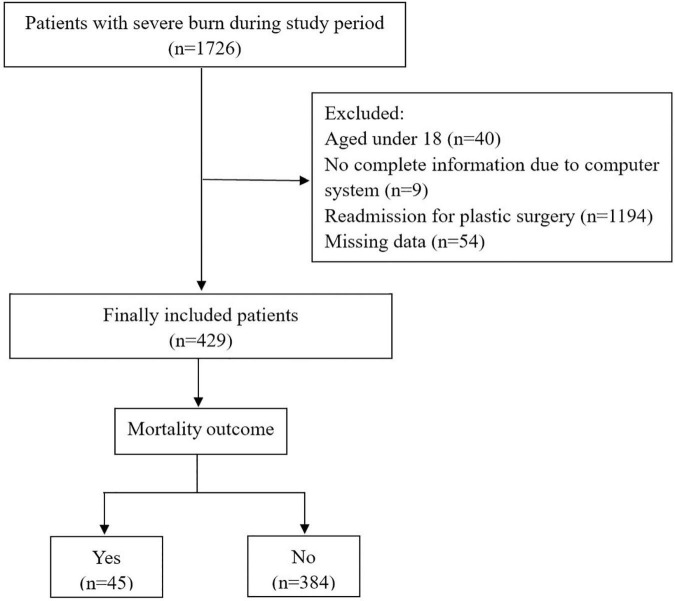
Flow diagram of study participants.

A total of 429 patients were eventually enrolled in present study and 28-day mortality was 10.49% (*n* = 45). A flow diagram of patient selection is shown in Figure 1. Of those included, there were 313 males (73%) and 116 females (27%), with a median age of 45 (34–55.5) years old. The type of burn of most patients were fire (78.6%). The demographic information and clinical data are detailed in Table 1. There were no significant differences in gender, type of burn, and comorbidities between 28-day mortality group and survival group (*P* > 0.05). The age, TBSA, APACHE II score, and SOFA score within 24 h of admission to the ICU were significantly higher in the 28-day mortality group than in the survival group (*P* < 0.01).

**TABLE 1:** Demographic information and clinical data (*N* = 429).

Variables	Overall (*n* = 429)	Non-survival (*n* = 45)	Survival (*n* = 384)	*P*-value
Age (year), median (IQR)	45 (34–55.5)	52 (39.5–60.5)	44 (33–54.8)	0.009
Male, *n* (%)	313 (73)	33 (73.3)	280 (72.9)	0.953
Type of burn, *n* (%)				0.465
Scald	46 (10.7)	3 (6.7)	43 (11.20)	
Fire	337 (78.6)	40 (88.9)	297 (77.3)	
Chemical	34 (7.9)	2 (4.4)	32 (8.3)	
Electric	12 (2.8)	0 (0)	12 (3.1)	
Any comorbidities, *n* (%)	79 (18.4)	8 (17.8)	71 (18.5)	0.907
TBSA%, median (IQR)	51 (37–75)	90 (75–95)	50 (36–70)	<0.001
APACHE II, median (IQR)	10 (9–12)	14 (10.5–18)	10 (9–12)	<0.001
SOFA, median (IQR)	3 (2–6)	8 (6–10.5)	3 (1–5)	<0.001
Inhalation injury (%)	125 (29.1)	25 (55.6)	100 (26.0)	<0.001

Data are expressed as median (interquartile range) for continuous variables and number (%) for categorical variables.

TBSA, total body surface area; APACHE, Acute Physiology and Chronic Health Evaluation; SOFA, Sequential Organ Failure Assessment.

For nutrition-related variables, there are significant differences in NRS2002, mNUTRIC, BMI, albumin, and pre-albumin in the outcomes of 28-day mortality (Table 2). The proportion of patients at nutrition risk (NRS2002 score ≥5; mNUTRIC score ≥1) were significantly higher in the 28-day mortality than in the survival group (*P* < 0.001). Albumin and pre-albumin on admission were significantly lower in the 28-day mortality group than in the survival group (*P* < 0.001).

**TABLE 2:** Nutrition risk and laboratory nutritional parameters (*N* = 429).

Nutritional factors	Overall (*n* = 429)	Non-survival (*n* = 45)	Survival (*n* = 384)	*P*-value
NRS2002				0.002
At risk, *n* (%)	88 (20.5)	17 (37.8)	71 (18.5)	
mNUTRIC				<0.001
At risk, *n* (%)	224 (52.2)	41 (91.1)	183 (47.7)	
BMI (kg/m^2^), median (IQR)	23.7 (21.3–25.8)	22.5 (20.1–24.9)	23.8 (21.5–25.9)	0.028
Albumin (g/L), median (IQR)	33 (28–37)	29 (24–32.5)	33 (28–38)	<0.001
Pre-albumin (g/L), median (IQR)	194 (147–237)	151 (101–191.5)	199 (154–243.8)	<0.001
NLR, median (IQR)	14.5 (9.8–20.7)	13.2 (8.9–20.8)	14.7 (9.8–20.8)	0.516
Hemoglobin (g/L), median (IQR)	165 (147–183)	166 (147–188.5)	165 (147–182)	0.558

Data are expressed as median (interquartile range) for continuous variables and number (%) for categorical variables.

NRS2002, Nutrition Risk Screening 2002; mNUTRIC, the modified Nutrition Risk in Critically ill; BMI, body mass index; NLR, neutrophil-to-lymphocyte ratio.

### Spearman correlation analysis

In Table 3, Spearman correlation analysis indicated that the NRS2002 score, nutritional status score and disease status score were positively correlated with TBSA and inhalation injury (*P* < 0.01), and negatively correlated with albumin and pre-albumin, respectively (*P* < 0.01). Regarding mNUTRIC, correlation analysis showed that the mNUTRIC score, APACHE II score, and SOFA score in the mNUTRIC scale were positively correlated with TBSA and inhalation injury, respectively (*P* < 0.01), negatively correlated with albumin and pre-albumin, respectively (*P* < 0.01).

**TABLE 3:** Correlations of the NRS2002 and mNUTRIC to clinical characteristics and nutritional indicators.

	TBSA	Inhalation injury	Albumin	BMI	Pre-albumin	Hemoglobin
NRS2002 score	0.443**	0.226**	−0.661**	−0.190**	−0.534**	–0.014
Disease status score	0.303**	0.203**	−0.235**	–0.042	−0.191**	0.078
Nutritional status score	0.372**	0.140**	−0.766**	−0.234**	−0.589**	–0.094
mNUTRIC score	0.316**	0.286**	−0.281**	–0.01	−0.233**	–0.05
APACHE II score	0.355**	0.249**	−0.286**	–0.058	−0.225**	0.097*
SOFA score	0.512**	0.336**	−0.41**	–0.044	−0.387**	0.09
Age	0.009	0.01	–0.079	0.074	–0.079	−0.149**
Number of comorbidities	–0.046	–0.53	0.057	0.141**	0.047	–0.035

*P < 0.05, **P < 0.01.

Spearman’s correlation coefficients were used for NRS2002, mNUTRIC, TBSA, inhalation injury, albumin, BMI, pre-albumin, and hemoglobin. NRS2002, Nutrition Risk Screening 2002; TBSA, total body surface area; mNUTRIC, the modified Nutrition Risk in Critically ill; BMI, body mass index; APACHE, Acute Physiology and Chronic Health Evaluation; SOFA, Sequential Organ Failure Assessment.

### The receiver operating characteristic curves of Nutrition Risk Screening 2002 and Modified Nutrition Risk in Critically ill

**FIGURE 2:**
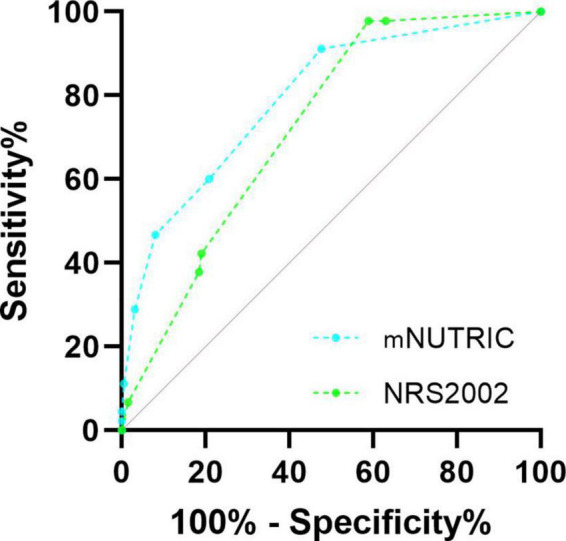
Receiver operating characteristic curves of NRS2002 and mNUTRIC to predict 28-day mortality in severely burned patients. The AUC for the NRS2002 was 0.726 (95% CI = 0.662–0.789), the AUC for the mNUTRIC was 0.795 (95% CI = 0.726–0.864). ROC, receiver operating characteristic; AUC, area under ROC curve; NRS2002, Nutrition Risk Screening 2002; mNUTRIC, the modified Nutrition Risk in Critically ill.

The ROC curves plotted to predict 28-day mortality are presented in the Figure 2, the area under the ROC curve (AUC) for the NRS2002 was 0.726 (95% CI = 0.662–0.789), and the AUC for the mNUTRIC was 0.795 (95% CI = 0.726–0.864). Jordan index was calculated by sensitivity and specificity, with all cut-off values of mNUTRIC previously reported in the previous literature performing poorly in this study population, as shown in Table 4. The new cut-off value mNUTRIC ≥1 showed the best sensitivity and specificity for predicting 28-day mortality, with a sensitivity of 91.1% and a specificity of 52.3%.

**TABLE 4:** Sensitivity and specificity according to different cut-off values for 28-day mortality.

Cut-off points	Sensitivity (%)	Specificity (%)	Youden
1	91.1	52.3	0.435
2	60.0	79.2	0.392
3	46.7	91.9	0.386
4	28.9	96.9	0.258
5	11.1	99.5	0.106

### Kaplan–Meier curves for overall survival

**FIGURE 3:**
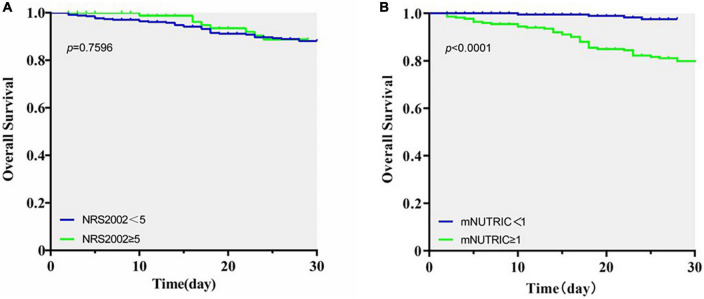
Kaplan–Meier survival curves in severely burned patients. **(A)** Overall survival based on NRS2002 scores. **(B)** Overall survival based on mNUTRIC scores.

Kaplan–Meier curves showed that there was no significant differences in 28-day overall survival (OS) rates between patients with high nutrition risk identified by NRS2002 and those with low nutrition risk (*P* = 0.760, Figure 3A). The survival probability of patients with mNUTRIC ≥1 was significantly lower than that with mNUTRIC <1 (*P* < 0.001, Figure 3B).

### Multivariate Cox regression analyses for 28-day mortality

In Table 5, we used univariate and multivariate COX regression analyses to identify independent predictors of 28-day mortality. We excluded parameters that were components of NRS2002 (age, APACHE II, and albumin) in model 1, and excluded age, APACHE II, SOFA, and albumin in model 2 respectively to avoid collinearities. In model 1, after adjusting for covariates, high NRS2002 was not independent predictors for 28-day mortality. TBSA (HR = 1.052, 95% CI = 1.028–1.077, *P* < 0.001) and SOFA (HR = 1.176, 95% CI = 1.072–1.291, *P* = 0.001) were independent predictors. In model 2, the multivariate Cox regression showed that patients with high mNUTRIC (HR = 4.265, 95% CI = 1.469–12.380, *P* = 0.008) had a significant higher probability of 28-day mortality. The possibility of 28-day mortality increased by 5.6% as the TBSA of severely burned patients increased by 1 point (HR = 1.056, 95% CI = 1.033–1.079, *P* < 0.001).

**TABLE 5:** Univariate and multivariate Cox regression analysis of risk factors for 28-day mortality in severely burned patients.

Variables	Univariate analysis	*P*-value	Model 1	*P*-value	Model 2	*P*-value
						
	HR (95% CI)		HR (95% CI)		HR (95% CI)	
**Age**	1.037 (1.015–1.058)	0.001				
**Male sex**	0.955 (0.492–1.854)	0.892				
**Type of burn**		0.118				
Fire	1					
Scald	0.495 (0.151–1.624)	0.246				
Chemical	0.446 (0.108–1.846)	0.265				
Electric	0.000 (0.000–1.213E + 289)	0.972				
**Any comorbidities**	0.995 (0.442–2.062)	0.906				
**TBSA%**	1.073 (1.051–1.095)	<0.001	1.052 (1.028–1.077)	<0.001	1.056 (1.033–1.079)	<0.001
**APACHE II**	1.180 (1.125–1.237)	<0.001				
**SOFA**	1.311 (1.229–1.398)	<0.001	1.176 (1.072–1.291)	0.001		
**Inhalation injury**	3.345 (1.842–6.074)	<0.001	1.313 (0.661–2.609)	0.437	1.482 (0.791–2.777)	0.219
**At nutrition risk (NRS2002)**	2.178 (1.189–3.991)	0.012	1.391 (0.239–2.609)	0.367		
**At nutrition risk (mNUTRIC)**	9.787 (3.505–27.323)	<0.001			4.265 (1.469–12.380)	0.008
**BMI**						
<18.5 kg/m^2^	2.044 (0.781–5.349)	0.145				
18.5–23.9 kg/m^2^	1					
24–27.9 kg/m^2^	0.597 (0.300–1.189)	0.142				
≥28 kg/m^2^	0.553 (0.167–1.833)	0.333				
**Albumin**	0.932 (0.892–0.974)	0.002				
**Pre-albumin**	0.990 (0.985–0.994)	<0.001	0.995 (0.990–1.000)	0.052	0.996 (0.992–1.001)	0.139
**NLR**	1.001 (0.980–1.022)	0.940				
**Hemoglobin**	1.000 (0.990–1.010)	0.962				

Model 1 was adjusted for risk factors including TBSA, SOFA, inhalation injury, and pre-albumin. The model 2 was adjusted for risk factors including TBSA, inhalation injury, and pre-albumin. HR, hazard ratio; TBSA, total body surface area; APACHE, Acute Physiology and Chronic Health Evaluation; SOFA, Sequential Organ Failure Assessment; NRS2002, Nutrition Risk Screening 2002; mNUTRIC, the modified Nutrition Risk in Critically ill; BMI, body mass index; NLR, neutrophil-to-lymphocyte ratio.

## Discussion

Our study is the first to compare the efficacy of NRS2002 and mNUTRIC in severely burned patients. After multivariable-adjusted Cox regression analyses, mNUTRIC was associated with adverse outcome. The NRS2002 is a nutrition risk screening tool based on 128 randomized controlled clinical trials promulgated by the Danish Association for Parenteral Enteral Nutrition (20). It is recommended by ESPEN (European Society for Parenteral and Enteral Nutrition) for screening nutrition risk in hospitalized patients. The threshold for the mNUTRIC remains controversial, with different studies using different cut-off values depending on the performance of the data (21, 22). In this present study, considering that the population in this study included severely burned patients admitted to the ICU after injury, and that the conversion score for the “days from admission to ICU” entry in mNUTRIC was 0, a new cut-off value was identified according to the Youden’s index.

The nutrition risk is common in severely burned patients and the incidence of nutrition risk in this study was 20.51% by NRS2002, 52.21% by mNUTRIC. This is much higher than the 12.81% reported in a previous study (23), probably because our study population was severely burned patients. Furthermore, considering that patients in this present study were directly admitted to the ICU after injury, the mNUTRIC cut-off value was set according to Youden’s index. Most studies about nutrient metabolism in burn patients focused on the pathophysiological mechanisms of hypermetabolism and methods for regulating metabolic responses (2, 24, 25), the nutritional status and nutrition risk of burn patients are not well understood. There are various nutrition risk screening scales such as NRS2002, Malnutrition Universal Screening Tool (MUST), NUTRIC, but severe burn victims usually endure multiple surgeries over an extended period of time and the risk of repeated infections, it is necessary to find a suitable screening tool for burn patients.

In this study, NRS2002 and mNUTRIC were significantly associated with clinical characteristics and nutritional indicators of burn patients. The total score of NRS2002 was positively correlated with TBSA (*P* < 0.01), and the total score of mNUTRIC, APACHE II, and SOFA was positively correlated with TBSA (*P* < 0.01). This suggests that NRS2002 and mNUTRIC may reflect clinical characteristics of burn patients. The mNUTRIC is a nutrition risk screening tool that takes into account disease severity. In univariate analysis, it was found that the levels of albumin and pre-albumin were associated with mortality (*P* < 0.01). Similar to the findings of a study, pre-albumin was a sensitive predictor of skin graft healing in burn patients (26). Pre-albumin has a short half-life of about 2 days, which can respond to acute changes in nutritional status (27) and is also used for monitoring nutritional therapy in burn patients (28). In multivariate analysis, TBSA was significantly associated with 28-day mortality. This is consistent with several foreign studies (29, 30). A previous study (31) also found that burn injury >40% TBSA was an independent predictor of mortality in burn patients (HR = 10.5, *P* < 0.01).

This present study illustrated the prognostic accuracy of mNUTRIC in severely burned patients. The mNUTRIC is more suitable than NRS2002 due to its better performance in screening nutrition risk and predicting 28-day mortality. Nutrition risk identified by mNUTRIC was significantly associated with 28-day mortality in severe burn patients (HR = 4.265, *P* = 0.008), suggesting that nutrition risk in severe burn patients has an important value in patients’ prognosis. Previous studies have reported that NRS2002 could serve as nutrition risk assessment tool in hospitalized patients, including cancer patients, stroke patients, and so on (32–36). But in present study, no significant association was found between high NRS2002 and 28-day mortality in multivariate analysis. This may be due to that the population in present study included severely burned patients admitted to the ICU, they underwent uncontrolled inflammatory responses and metabolic disturbances after injury. NRS2002 focus on pre-injury dietary and weight loss, and mNUTRIC takes disease severity into account. Besides, the parameters in mNUTRIC might also explain the association between nutrition risk with 28-day mortality. To our knowledge, only one study (23) validated the feasibility of mNUTRIC in ICU burn patients, but there is no literature yet comparing the ability of different nutrition risk screening tools in burn patients. Compared with the existing burn prognosis prediction models such as Baux and Ryan (37, 38), our study took into account that the body’s nutrient metabolism will undergo drastic and continuous changes after burns, and the nutrient reserve may be closely related to the patient’s condition changes after injury (1). On the one hand, we recommend mNUTRIC as nutrition risk screening tool to identify patients who would benefit more from nutrition therapy in severely burned patients. On the other hand, combining mNUTRIC with TBSA may more comprehensively reflect the severity of burn patients.

This study has some limitations. Firstly, it used a single-center retrospective study design, so the generalizability of the findings may be unfavorable. Secondly, although the treatment principles were the same, there may have been differences in the specific nutritional treatment regimen and timing, we did not assess the impact of adequate nutritional therapy on mortality. Clinical trials are needed in the future to explore whether nutritional therapy can improve outcomes in patients at nutrition risk. Thirdly, the assessment of NRS2002 and mNUTRIC was done by one investigator based on EMR, and it may be more accurate to use a prospective design to collect information in subsequent studies.

## Conclusion

Based on our results, mNUTRIC could serve as nutrition risk screening tool to identify patients who would benefit more from more aggressive nutritional therapy in severely burned patients. The mNUTRIC was associated with 28-day mortality in severely burned patients, and combined with TBSA, it may comprehensively determine the severity of patients.

## Data availability statement

The raw data supporting the conclusions of this article will be made available by the authors, without undue reservation.

## Ethics statement

The studies involving human participants were reviewed and approved by the Research Ethics Committee of Ruijin Hospital, Shanghai Jiao Tong University School of Medicine (Decision No. 2022019). Since this present study was retrospective, patients were not required to provide written informed consent.

## Author contributions

ZM: study concept and design, data collection, statistical analysis, and writing—original draft. YZ: resources, statistical analysis, and writing—original draft. QZ: resources, guidance, supervision, and editing the manuscript. BW: study concept and design, editing the manuscript, guidance and supervision. All authors read and approved the final manuscript.
